# Thermal Processing for the Release of Phenolic Compounds from Wheat and Oat Bran

**DOI:** 10.3390/biom10010021

**Published:** 2019-12-22

**Authors:** Lavinia Florina Călinoiu, Dan Cristian Vodnar

**Affiliations:** Faculty of Food Science and Technology, Institute of Life Sciences, University of Agricultural Sciences and Veterinary Medicine Cluj-Napoca, Calea Mănăștur 3-5, 400372 Cluj-Napoca, Romania; lavinia.calinoiu@usamvcluj.ro

**Keywords:** thermal processing, cereal bran, phenolic compounds, bioactivity, circular economy

## Abstract

The aim of the present paper was to identify the major polyphenolic compounds and investigate the antioxidant, antimutagenic, and antimicrobial activities of industrially-derived cereal byproducts—wheat bran (WB) and oat bran (OB)—before (fresh) and after thermal processing (TP) (10 min, 80 °C), coupled with ultrasound-asssited extraction. The results showed that the thermal process improved the total phenolic content of WB by +22.49%, and of OB with +25.84%. After the TP, the phenolic concentration showed a significant relative percentage increase in the case of WB (ferulic acid +39.18%, vanillic acid +95.68%, apigenin–glucoside +71.96%, *p*-coumaric acid +71.91) and of OB (avenanthramide 2c +52.17%, dihydroxybenzoic acids +38.55%). The best antioxidant capacity was registered by OBTP followed by WBTP. The strongest antimicrobial inhibition was attributed to the WBTP sample. Both thermally processed matrices had strong antimutagenic activity toward *S. typhimurium* TA100. This thermal processing was tested on bran based on its practical application within the food industry, considering the design of different cereal byproducts derived from functional foods and nutraceuticals.

## 1. Introduction

From ancient times, cereals have represented the base nourishment in human nutrition all over the world. Wheat and oat represent staple foods for the worldwide population [1,2]. The European Flour Millers report [3] from 2016 pointed out that in Europe, more than 45 million tons/year of wheat and oat are processed, generating more than 6.5 million tons of bran that are further used for animal feeding. Byproducts, such as bran, are abundant in valuable compounds [4] (e.g., dietary fibers, minerals, vitamins, polyphenols, or phytosterols), with a positive impact on health [2,5].

Lately, more research groups have focused on the exploitation of particular wastes and byproducts for human consumption by extracting and intensifying the bio-accessibility of the existing bioactive nutrients [6,7,8,9,10,11,12,13,14,15]. The major bioactive compounds of wheat and oat brans are the phenolic acids and flavonoids groups. Among phenolic acids, the most representative ones are ferulic acid, dihydroxybenzoic acid, sinapic acid, *p*-coumaric acid, and avenanthramide [16,17]. Avenanthramides are naturally-derived oat phenolic compounds with very good antioxidant activity [18]. Phenolic compounds present in wheat and oat bran are insoluble, being bound to the cellulose and hemicellulose structures [19,20,21], making the conventional extraction processes very difficult. Expensive, time-consuming, and unecological procedures like chemical and enzymatic hydrolysis or mechanical crash have been applied over time as pretreatment methods for the release of the phenolic compounds from bran [16,22,23].

The thermal processing of bran is one of the most practical methods that increase its beneficial effects on human health [1,24,25]. Thermal processing enhances bran’s solubility by making the micronutrients bio-available [26], reducing their complex structure [27,28], and decreasing the content of some inhibitory factors [1]. Previous studies on cereal products have presented that thermal processing contributes to the cell wall and cellular constituents’ breakdown through the release of the bound phenolic acids [29].

There are also novel extraction technologies for making accessible the valuable compounds found in vegetal matrices [30]. For example, ultrasound-assisted extraction (UAE) [31,32] represents a novel and low-cost technique that can improve the bioactive compound extraction rate and efficiency. UAE can be applied for both the extraction of interest compounds and for their encapsulation by different biopolymers [33,34,35,36,37]. There are different thermal processes (e.g., steaming, pressured steam-heating, drum drying, roasting, and microwave heating), largely applied to increase the palatability, stability, and safety of cereal-based food products. Few of the mentioned methods boost the extractability of the phenolics in specific materials [24,38,39]. Blanching was reported to increase the extraction of bioactive compounds by its softening attribute [1,40]. Extrusion might positively or harmfully influence the phenolic compounds’ bioaccessibility from brans. High temperature can induce the degradation of the phenolics that are heat labile, and can also generate the polymerization of some phenolics under elevated pressure in extrusion cooking [26].

The utilization of industrially-derived cereal byproducts may represent an important source of nutrients, while its reutilization can provide a significant source of income. To the best of our knowledge, the bioactive potential of wheat and oat bran after being thermally treated at 80 °C for 10 min was not previously reported, whereas the antimutagenic and antimicrobial activities of the extracts are of significant novelty. Considering all this, the present research focused on investigating the polyphenolic content and composition, and the biological activity potential via antioxidant, antimicrobial, and antimutagenic capacities of wheat and oat bran, before and after the thermal treatment. This thermal processing was chosen for its high utility within the food industry (e.g., pasteurization, blanching), considering the design of different cereal byproducts derived from functional foods and nutraceuticals.

## 2. Materials and Methods

### 2.1. Chemicals and Materials

The white wheat bran (WB) and oat bran (OB) (Solaris, Romania), commercially available on the local market, were used for present experiments. Firstly, they were defatted thrice with hexane (1:5 *w*/*v*), for 5 min at room temperature while stirring, followed by filtration, and were then allowed to dry for 24 h at room temperature. The bran material obtained was immediately used to extract the bioactive compounds (detailed in Section 2.2.), and was considered further as fresh (F) samples, precisely fresh wheat bran (WBF) and fresh oat bran (OBF) samples. The same bran materials were further used to make the thermal samples (TP), precisely the thermally processed wheat bran (WBTP) and thermally processed oat bran (OBTP), by mixing each type of bran with water (5:1 *v*/*w* for wheat bran and 2:1 *v*/*w* for oat bran) and were thermally processed (10 min at 80 °C), followed by their extraction (detailed in Section 2.2.).

Folin–Ciocalteu’s phenol reagent, DPPH (1,1-diphenyl-2-picrylhydrazyl), sodium carbonate, acetonitrile, acetic acid, and methanol were obtained from Sigma-Aldrich (Steinheim, Germany). The Mueller–Hinton agar, thioglycollate broth with resazurin, and Mueller–Hinton broth were obtained from BioMerieux (Craponne, France).

### 2.2. Ultrasound-Assisted Extraction (UAE)

The fresh (WBF and OBF) and thermally processed samples (WBTP and OBTP) were individually extracted three times with 80% methanol (1:5 *w*/*v*) at 40 °C for 1 h in an ultrasonic bath (Elmasonic E15H, Elma, Singen, Germany) [6,27]. After centrifugation (4000× *g* for 10 min), the supernatants were filtered, and then concentrated to dryness. The dried extracts were reconstituted in methanol and stored (4 °C) until analysis (total phenolic content (TPC) assay and HPLC analysis of individual phenolic compounds, as well as antioxidant, antimutagenic, and antimicrobial activities). The results were calculated based on dry matter.

### 2.3. Total Phenolic Content Analysis

The TPC was evaluated via the Folin–Ciocalteu method [27,41,42]. From each type of extract, a quantity of 25 µL was combined with 125 µL of Folin–Ciocalteu reagent (0.2 N) and allowed to stand for 2 min at room temperature. Afterward, on top of the mixture was added 100 µL of 7.5% (*w*/*v*) Na_2_CO_3_ solution. The obtained solution was incubated at 25 °C in the dark for 2 h. Methanol represented the blank and the absorbances were registered at 760 nm. In the preparation of the standard curve gallic acid (0.01–1.00 mg/mL) was used, and the TPC in the samples was reported as gallic acid equivalents (GAE) in mg/100 g dry weight (DW) of bran. This analysis was repeated three times for each extract.

### 2.4. HPLC–DAD–ESI–MS Identification of the Phenolic Composition

The characterization and identification of the individual phenolic compounds were done using an 1200 HPLC Agilent system connected to a DAD detector and an Agilent 6110 single-quadrupole MS-detector. The phenolic compound separation was conducted at 25 °C on a XDB C18 Eclipse column (4.6 × 150 mm, 5 µm) (Agilent Technologies, Santa Clara, CA, United States). Two solvents consisting of 0.1% acetic acid/acetonitrile (99:1) in distilled water (*v*/*v*) (solvent A) and 0.1% acetic acid in acetonitrile (*v*/*v*) (solvent B) were used. The flow rate was set on 0.5 mL/min, according to Dulf et al.’s [41] gradient elution program. The phenolic components were established by a comparison of their retention times, UV visibility, and mass spectra with reference standards. The MS fragmentation was based on the ESI (+) module, scanning between 100 and 1000 *m*/*z*, at 300 °C, with a nitrogen flow of 7 L/min and a capillary voltage of 3000 V. The absorbance spectra was in the range of 200–600 nm, and the eluent was being monitored by DAD. The detection was registered at 280 nm and 340 nm. Data were analyzed by using Agilent ChemStation Software (Rev B.04.02 SP1, Palo Alto, California, United States). For the quantitative determination of the phenolic compounds, a calibration curve with gallic acid was used to quantify hydroxybenzoic acids (μg/g DW) (*r*^2^ = 0.9959) and a calibration curve with ferulic acid for the quantification of hydroxycinnamic acids (μg/g DW) (*r*^2^ = 0.9959).

### 2.5. DPPH Free Radical Scavenging Capacity

The free radical scavenging ability of the UAE methanolic extracts of fresh and thermally processed samples was measured according to a previously reported method [43,44]. A BIOTEK spectrometer (515 nm) (BioTek® Instruments, Inc., Winooski, VT, USA) was used to monitor the reaction between DPPH and antioxidant compounds present in the samples. The antioxidant capacity of the samples expressed as the equivalent factor F (mM Trolox) was calculated at 100 g DW. The inhibition percentage (I%) followed the equation

[1 − (test sample absorbance/blank sample absorbance)] × 100.(1)

### 2.6. Antibacterial Activity

This bioassay involved five bacterial strains: two Gram-positive bacteria (*Staphylococcus aureus* (ATCC 49444) and *Enterococcus faecalis* (ATCC 29212)) and three Gram-negative bacteria (*Pseudomonas aeruginosa* (ATCC 27853), *Salmonella typhimurium* (ATCC 14028), and *Escherichia coli* (ATCC 25922)). The strains used were obtained from the Food Biotechnology Laboratory, University of Agricultural Sciences and Veterinary Medicine of Cluj-Napoca (UASVM CN), Romania. The Mueller–Hinton agar was used for aerobic bacteria strains and cultures were stored at 4 °C (monthly subcultured).

The previously described microdilution technique [11,27] was used to evaluate antimicrobial activity. The overnight cell suspensions were adjusted with a sterile saline solution to a concentration of approximately 2 × 10^5^ CFU/mL in a final volume of 100 μL per well. The inoculum was stored at 4 °C for further use. The serial dilution technique using 96-well plates was used to determine the minimum inhibitory concentrations (MICs). Different dilutions of the cereal bran extracts were performed by adding 100 μL of Mueller–Hinton broth, and afterward, 10 μL of inoculum to all the wells, followed by incubation for 24–48 h at 37 °C. The MIC of the samples was measured by the change from blue to pink of added resazurin solution after incubation for 2 h at 37 °C. This change indicates a reduction of resazurin, and therefore, bacterial growth. The MIC was defined as the lowest concentration that prevented this color change. The positive control used was streptomycin (Sigma P 7794, Santa Clara, CA, United States) (0.05–3.00 mg/mL), whereas water represented the negative control.

### 2.7. Mutagenic and Antimutagenic Assay

Mutagenic and antimutagenic activity of fresh (WBF, OBF) and thermally processed (WBTP, OBTP) samples were evaluated by using the plate incorporation method previously described by Marton and Ames [45] and Sarac and Sen [46]. The following direct mutagens, precisely 4-nitro-o-phenylenediamine (4-NPD, 3 µg/plate) and sodium azide (NaN_3_, 8 µg/plate) represented the positive controls for *S. typhimurium* TA98 and *S. typhimurium* TA100 (Food Biotechnology Laboratory, UASVM CN, Romania). The negative control was represented by ethanol/water (1:1, *v*/*v*). The concentration of extracts used was 5 mg/plate. The antimutagenicity was calculated according to the previously reported [47] formula:

% Inhibition = [1 − *T*/*M*] × 100 (2)

Whereas, *T* represents the number of revertants per plate in the presence of the mutagen and extract, and *M* is the number of revertants per plate in the absence of extract. The assay was done in triplicate, and the data is reported as the mean ± standard deviation (SD). The antimutagenicity interpretation is strong: 40% or more inhibition; moderate: 25–40% inhibition; and low/none: 25% or less inhibition [48].

### 2.8. Statistical Interpretation

All tests were conducted in triplicate, and data is reported as the mean ± standard deviation (SD). The statistical differences between extracts were performed using GraphPad Prism Version 8.0.1 Student’s *t*-test (Graph Pad Software Inc., San Diego, CA, United States). Differences between means at the 5% level were considered statistically significant.

## 3. Results and Discussions

### 3.1. Evaluation of Total Phenolic Content

**Figure 1:**
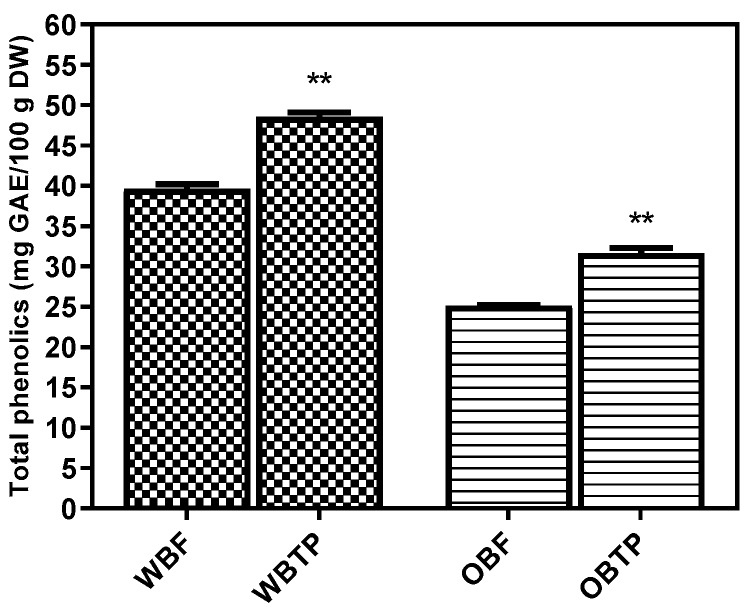
Total phenolic content (Folin–Ciocalteu method) of the wheat and oat bran extracts (fresh and thermally processed). The total phenolic content of the extracts is expressed as gallic acid equivalents (GAE) in mg/100 g dry weight (DW). Values are expressed as mean values ± standard deviation (SD), *n* = 3, and are followed by symbols (**) indicating significant differences (*p* < 0.05) between samples (Student’s *t*-test; GraphPad Prism Version 8.0.1, Graph Pad Software, Inc., San Diego, CA, United States).

The TPC of fresh and thermally processed cereal byproducts extracts using the Folin–Ciocalteu method is presented in Figure 1 below, for each type of bran. The UAE enhanced the phenolic release. According to a related previous study [49], the TPC of ultrasound extraction (25 min) of wheat was 3.12 ± 0.03 mg gallic acid equivalents (GAE)/g bran.

Significant differences (*p* < 0.05) between the TPCs before and after the thermal processing of bran materials were registered. The thermally processed samples of wheat bran had a higher TPC (48.52 ± 0.55 mg GAE/100 g dry weight), and OBF had the lowest (25.15 ± 0.45 mg GAE/100 g dry weight). The phenolic content for each type of bran showed significantly (*p* < 0.05) higher values (48.52 ± 0.55 mg GAE/100 g DW for WBTP, and 31.65 ± 0.59 mg GAE/100 g DW for OBTP) in thermally processed than in fresh samples (39.61 ± 0.51 mg GAE/100 g dry weight for WBF and 25.15 ± 0.45 mg GAE/100 g dry weight for OBF). Therefore, the thermal processing may enhance the phenolic release in the extracts, a fact which can be explained considering that the extraction of intracellular contents is improved by thermal processing. Moreover, Wang, et al. [39] found that high temperatures enhanced the phenolic content due to the hydrolysis of polysaccharides. In addition, Sharanappa et al. [50] studied the bioactive compounds in eight wheat bran cultivars, whereas the total phenolic acids ranged between 43.07 ± 6.4 and 74.61 ± 8.3 mg GAE/100 g, which is in accordance with our findings. In the study of Li et al. [25], the TPC of heat-treated purple wheat bran ranged between 3.68–6.98 mg/g, depending on the type of solvent, which is slightly higher than levels found in untreated purple wheat bran extracts (3.34–5.98 mg/g). Therefore, their findings are in agreement with our results. Brindzová et al. [51] reported a total phenolic content in the studied wheat bran extract of 2.7 mg/g (expressed as gallic acid equivalent), whereas the total flavonoid content, expressed as a rutin equivalent, was 70.8 μg/g. According to Bryngelsson et al. [38], autoclaving can more effectively increase the solvent-extractable phenolic compounds—predominantly *p*-coumaric acids and ferulic content—than the steaming method. Recent literature has reported that wheat kernel hydrothermal processing increased the phenolic acid content’s accessibility [1]. The study by Chandrasekara et al. [52] stated that in five millet grains, through hydrothermal treatment (boiling for 30 min), most of the phenolic compounds content were constant, while a diminished total phenolic content was observed in fingers (local), at 36%, and dehulled fingers (Ravi) at 11%. Depending on the employed assay method and type of cereal, each hydrothermal treatment may have different impacts on the antioxidant activity. In cereal grains, due to the varying thermal treatment, the phenolic content in the grains can be associated with the grain nature, type, and location of the phenolic compounds, as well as heat treatment duration and severity [39].

### 3.2. Evaluation of Antioxidant Capacity

**Figure 2:**
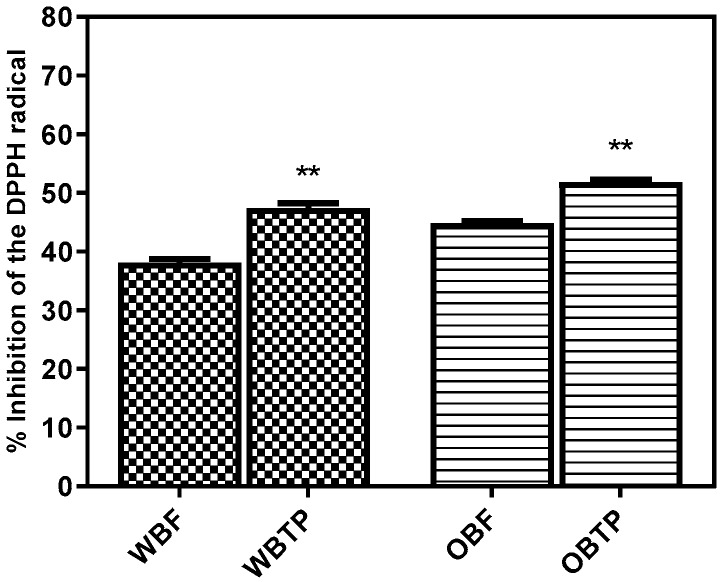
Antioxidant activity (DPPH free radical scavenging assay) of the wheat and oat bran extracts (fresh and thermally processed). The percentage inhibition (*I*%) was calculated as [1 − (test sample absorbance/blank sample absorbance)] × 100. Values are expressed as mean values ± SD, *I*%, *n* = 3, and are followed by different symbols (**) indicating significant differences (*p* < 0.05) between samples (Student’s *t*-test (*p* = 0.05); GraphPad Prism Version 8.0.1, Graph Pad Software, Inc., San Diego, CA, United States).

The antioxidant capacity of the samples was measured by the DPPH method, and the changes are illustrated in Figure 2. After thermal treatment, wheat bran (+24.41%) and oat bran (+15.88%) registered statistically significant increases (*p* < 0.05). Compared to WBF, OBF showed a higher antioxidant capacity, probably due to the presence of increased avenanthramide content; avenanthramides have been previously reported for their strong scavenging capacity [6]. OBTP extract registered the highest antioxidant activity, followed by WBTP extract. Interestingly, WBTP extract registered the highest relative percentage increased, which was ascribed to the high increase of a specific subclass of flavonoid, apigenin–glucoside (+71.96%), as well to the large increase of ferulic acid (+39.18%). In addition, flavonoids were reported to have higher antioxidant activities than vitamins C and E and carotenoids [53]. The increase in antioxidant activities of thermally processed oat bran showed that the antioxidant activity might be enhanced also by other polyphenols, such as avenanthramides, unique oat-specific compounds that increased significantly during the thermal treatment. These findings are in agreement with the results of Dewanto et al. [29], who reported that the total antioxidant activity of sweet corn was elevated by 44% after thermal processing. Also, the antioxidant activity can be heightened after several autoclaving cycles, which points out the possibility that the decompression at the end of autoclavation has a decisive role in the breakdown of the cell wall, due to the fast gas expansion dissolved through pressurization [54].

### 3.3. Evaluation of Phenolic Composition by HPLC-DAD-ESI-MS

The UAE of phenolic compounds upon the thermal treatment improved the release of polyphenolic compounds like ferulic acid and apigenin glucoside in WB, and avenanthramides in OB. There were identified a total of 10 phenolic compounds belonging to the following phenolic classes/subclasses: hydroxycinnamic (ferulic acid, caffeic acid, *p*-coumaric acid, sinapic acid, avenanthramide 2c, avenanthramide 2p, and avenanthramide 2f) and hydroxybenzoic (vanillic and dihydroxybenzoic) acids, as well as flavones (apigenin–glucoside). The phenolic composition of both wheat and oat bran are illustrated in Table 1 below.

#### Changes in Phenolic Composition After the Thermal Process

**Figure 3:**
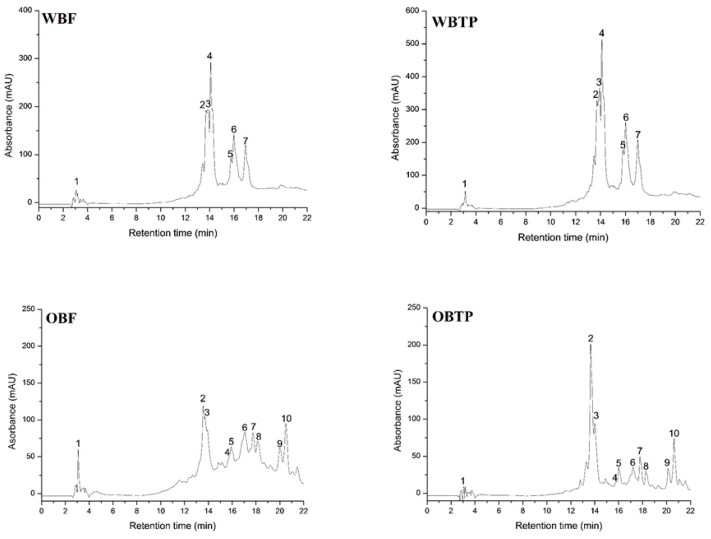
HPLC chromatograms of WBF, WBTP, OBF, and OBTP.

The changes in the phenolic compounds of WB and OB after thermal processing are illustrated in Table 2 and Table 3, respectively, reported as μg GAE/g DW. The changes in all individual phenolic acids are also reported in Figure 3 via the HPLC chromatograms.

The phenolic composition of WB (Table 2) improved after thermal treatment, and each individual phenolic compound had a significant relative percentage increase versus fresh samples (ferulic acid +39.18%, caffeic acid +68.15; vanillic acid +95.68%, dihydroxybenzoic acid +31.54%, apigenin-glucoside +71.96%; *p*-coumaric acid +71.91, sinapic acid +49.65). The major phenolic acid identified was dihydroxybenzoic acid. The second major phenolic compound identified was ferulic acid, followed closely by apigenin–glucoside, having an almost twofold increase after the thermal process. The high relative percentage increase in the antioxidant activity of WBTP may be attributed to the increased content of this compound reported having a good antioxidant capacity [55,56,57], as well as *p*-coumaric and sinapic acid presence.

According to a previous study [58], in wheat flour, after heat stress (100 °C), due to the degradation of the conjugated polyphenolic compounds (e.g., tannins), an increase of several phenolic compounds (vanillic, ferulic, *p*-coumaric, syringic or simple phenolics) was obtained. The thermal processing method can discharge unavailable phenolics, which can accumulate in the cellular vacuoles. The operating conditions of the process also influence the phenolic compound content variation. For example, the discharge of phenolic compound moisture content is very reliant on the temperature, time, and moisture content throughout the extrusion processing [59].

All the phenolic acids identified in OB before and after the thermal process are reported in Table 3. Among the OB identified compounds are the avenanthramides. Dihydroxybenzoic acids were also a major compound identified with a higher concentration (76.278 ± 1.140 μg/g) after the thermal process. The avenanthramide 2c had the highest relative percentage increase among avenanthramides (+52.17%), followed by 2f (+16.69% vs. fresh). The other phenolic acids identified were vanillic, *p*-coumaric, sinapic, and ferulic acids, with, higher percentage increases of +22.59%, +16.28%, +13.06%, and +5.88%, respectively, registered after thermal processing. There are studies that have reported the caffeic and ferulic acids as main phenolic compounds in oat [60]; vanillic, sinapic, *p*-coumaric, and protocatechuic acid have been found as well [61,62]. Our findings are in agreement with previously reported phenolic compounds in oat bran [63], being vanillic, p-hydroxybenzoic, ferulic, *p*-coumaric, caffeic, and sinapic acids. According to the study of Dimberg et al. [64], the increase of avenanthramides might be explained by a release of bound forms, increasing extractability after processing, or a combination of these factors.

A related literature study [38] presented the autoclavation process of oat grains, which increased the release of the different phenolic acids in a different way. The highest growth was observed in *p*-coumaric acid at 1137%, vanillin at 1044%, and ferulic acid at 222%, whereas the caffeic acid content diminished to nondetectable levels. The growth alteration is most probably due to particular linkages and the position between the parts of the cell wall and phenolic acids. The predominant phenolic fraction in oat groat is caffeic acid, and in the hull fraction ferulic acid, sinapic acid, and *p*-coumaric acids are dominant [39]. The same study reported that the significant growth of ferulic and *p*-coumaric acids showed that the covalent linkages between the fiber components and hydroxycinnamic acids could be broken through autoclaving, while due to caffeic acid’s heat-sensitive nature, this component is nearly entirely degraded after this process. As a consequence of ferulic acid’s thermal degradation to 4-vinylguaiacol and being oxidized additionally to vanillic acid, the literature has reported an increase of vanillic content [65], which is in line with our findings.

### 3.4. Identification of Antibacterial Inhibition

The studied cereal brans proved to have an antimicrobial effect against tested bacteria. The findings are presented in Table 4. For *S. aureus*, an increased antibacterial effect was reported for OBTP, with an MIC of 0.9375 (mg/mL), followed by WBTP and OBF, with an MIC of 3.75 (mg/mL). These results may be explained considering the enhanced phenolic composition, such as avenanthramides in oat bran, and ferulic acid and apigenin–glucoside compounds in wheat bran. Previously studies have reported a range of phenolic compounds with different antimicrobial effects, considering their capacity to attack a variety of bacteria. The strong antioxidant capacity of thermally processed wheat bran and oat bran (considering the increased content of ferulic acid, apigenin-glucoside, and avenanthramides), may underline the possible correlation between the antimicrobial effect and antioxidant capacity exhibited by the presence of phenolic compounds. The *S. aureus* was the most sensitive bacterium to the tested extracts, a fact which was in agreement with our previous work [27,28]. WBTP registered an MIC of 1.875 (mg/mL) against *E. faecalis*, whereas OBTP showed an MIC of 3.75 (mg/mL) toward the same strains. The moderate antibacterial effect was found toward the Gram-negative bacterium, *P. aeruginosa*, by WBTP and OBTP, as well as WBF extracts with MICs of 3.75 (mg/mL) and 7.5 (mg/mL), respectively. Also, moderate antibacterial activity was exhibited by WBTP against *E. coli*, with an MIC of 3.75 (mg/mL); the bacteria was quite resistant against the other types of extracts. Regarding the Gram-negative bacteria, *S. typhimurius*, WBTP and OBTP extracts, considering their increased phenolic content and higher scavenging capacity, registered the highest antibacterial effects toward the tested bacteria (MIC = 7.5). Therefore, it may be stated that the thermally processed extracts exhibited antibacterial effects when compared to fresh extracts, towards the tested bacteria.

The highest antibacterial effect was exhibited by the thermally processed wheat bran extract, towards almost all the strains. When analyzing Table 4, Gram-positive bacteria were more sensitive when compared to Gram-negative ones.

### 3.5. Identification of Mutagenic and Antimutagenic Capacity

The fresh and thermally processed (80 °C, 10 min) wheat bran and oat bran samples were tested for their mutagenic and antimutagenic activity, based on our previous studies on their antioxidant activity [6]. The influence of the thermal process, as well as the type of cereal, had an important influence on the number of revertants in *S. typhimurium* TA98 and TA100. The fresh and thermally processed extracts from wheat and oat bran had no mutagenic effect, which was in line with the few previous studies [51,66]. The extract’s antimutagenicity potential towards *S. typhimurium* TA98 and TA100 is presented in Table 5. The antimutagenic effects of fresh and thermally processed cereal extracts (oat bran and wheat bran) were tested against direct-acting mutagens 4-NPD for TA98 and sodium azide (NaN_3)_ for TA 100.

With respect to *S. typhimurium* TA98, the tested samples proved to be a significant inhibition of the number of revertants of strain TA98 induced by 4-NPD. Therefore, strong antimutagenic activity for OB (both forms) was found. The higher inhibition was registered by the thermally processed sample, with inhibition of 57.43%. A possible explanation for this could be the fact that the thermal process increased scavenging capacity. A moderate inhibition was proved by WBTP and WBF, considering the percentages of 37.95% and 31.28%.

With respect to TA100, oat bran, with and without thermal processing, proved again to have a stronger antimutagenic capacity, whereas the best inhibition was exhibited by the thermally processed sample at precisely 67.32%, followed by OBF, WBTP, and WBF. Moreover, the WB samples inhibited the mutagenic effect of sodium azide more than 50%, whereas the thermally processed sample reach higher inhibition (58.84%). These favorable effects are probably associated with a high content of phenolic acids [67] and flavonoids [68] which markedly decrease the mutagenic activity of the standard mutagens examined. Moreover, to the best of our knowledge, this is the first study assessing the mutagenic and antimutagenic activities of thermally processed (80 °C, 10 min) wheat bran and oat bran.

Our findings are in agreement with previous studies, precisely the study of Brindzová et al. [66], who reported that extracts from oat inhibited the number of revertants of strain TA100 induced by 5NFAA by almost half, and wheat bran inhibited up to 80%. Also, the same study reported no significant influence of the extracts (oat, wheat bran, buckwheat) on the number of revertants of strain TA98 induced by 2-NF. Another study [51] reported favorable antimutagenic effects of the DMSO wheat bran extracts, precisely inhibited the number of revertants of strain TA100 induced by 5NFAA up to 70%. The differences in results can be explained by the solvent extract, by the direct mutagen used, and the specific method involved.

Considering our findings, wheat and oat brans have significant content of health-related compounds, precisely phenolic compounds, which may have an important role in the antioxidant and antimutagenic capacities of the tested extracts. The higher antimutagenic activity was reported toward the strain *S. typhimurium* TA100.

## 4. Conclusions

These findings are supportive arguments for the utilization of cereal processing industry byproducts that are good bioactive sources after thermal processing (10 min at 80 °C) in the food industry. To best of our knowledge, this is the first study investigating this thermal process on wheat and oat bran while analyzing its influence on antioxidant, antimicrobial, and antimutagenic activities.

The results showed that the thermal process improved the total phenolic content of wheat bran by 22.49%, and of oat bran by 25.84%. After the TP, the phenolic concentration showed a significant relative percentage increase in the case of both WB (ferulic acid +39.18%, vanillic acid +95.68%, apigenin-glucoside +71.96%; *p*-coumaric acid +71.91) and OB (avenanthramide 2c +52.17%, dihydroxybenzoic acids +38.55%). The best antioxidant inhibition percentage was registered by OBTP (51.75%), followed by WBTP (47.4%). Enhanced scavenging capacity of thermally processed samples sustains the idea that the tested thermal treatment may have a positive influence on antioxidant activity. Considering the antioxidant via radical scavenging activity of wheat bran and oat bran extracts, fresh and thermally processed, it may be stated that wheat/oat brans are a rich source of compounds with strong antioxidant effects, and as such may be used for the preparation of nutraceuticals or functional foods in the future.

Compared to the fresh samples, the thermally processed ones had a higher antibacterial effect towards the tested microorganisms, while strong inhibition capacity was registered towards *S. aureus* by thermally processed oat bran (MIC = 0.9375 mg/mL). The thermally processed oat bran had also a higher antimutagenic inhibition percentage towards *S. thyphimurium* TA98 (57.43%) and TA100 strains (67.32%).

The bioactive potential via phenolic compounds in industrial processing-derived cereal byproducts after a thermal treatment enables the bran part to be perceived as a significant source for the food industry, despite its actual high utility in animal feeding. By using these byproducts, an extra source of income may be derived for the cereal industry, while at the same time impacting the environment less, considering its emerging disposal problem. Moreover, this approach may represent a strong solution for feeding 10 billion people by 2050, sustaining the circular economy as well.

## Figures and Tables

**Table 1:** Identification of phenolic compounds in fresh/thermally processed samples of wheat bran (WB) and oat bran (OB).

Sample	Peak No.	Retention Time (min)	[M + H]^+^ (*m*/*z*)	UV λmax (nm)	Compound	Class/Subclass
WB	1	2.94	156	250	Dihydroxybenzoic acids	Hydroxybenzoic acid
	2	13.51	181,163	320	Caffeic acid	Hydroxycinnamic acid
	3	13.89	169	255, 300	Vanillic acid	Hydroxybenzoic acid
	4	14.31	433	272, 340	Apigenin-glucoside	Flavone
	5	15.78	165	319	*p*-Coumaric acid	Hydroxycinnamic acid
	6	16.85	225	321	Sinapic acid	
	7	17.12	195	322	Ferulic acid	
OB	1	2.94	156	250	Dihydroxybenzoic acids	Hydroxybenzoic acid
	2	13.51	181, 163	320	Caffeic acid	Hydroxycinnamic acid
	3	13.89	169	255, 300	Vanillic acid	Hydroxybenzoic acid
	4	15.78	165	319	*p*-Coumaric acid	Hydroxycinnamic acid
	5	16.85	225	321	Sinapic acid	
	6	17.12	195	322	Ferulic acid	
	7	17.97	316	330	Avenanthramide 2c	
	8	19.81	300	330	Avenanthramide 2p	
	9	20.37	330	330	Avenanthramide 2f	

**Table 2:** Phenolic compound analysis of the fresh and thermally processed wheat bran samples.

Sample	di-OH Benzoic Acid	Caffeic Acid	Vanillic Acid	Apigenin–Glucoside	*p*-Coumaric Acid	Sinapic Acid	Ferulic Acid
WBF	62.15 ± 0.20 ^b^	12.37 ± 0.05 ^b^	15.73 ± 0.07 ^b^	20.40 ± 0.11 ^b^	8.65 ± 0.02 ^b^	14.18 ± 0.08 ^b^	22.56 ± 0.13^b^
WBTP	81.75 ± 1.07 ^a^	20.81 ± 0.12 ^b^	30.788 ± 0.19 ^a^	35.09 ± 0.21 ^a^	14.88 ± 0.09 ^a^	21.23 ± 0.13 ^a^	31.41 ± 0.33 ^a^

Values (mean ± SD, μg/g DW, *n* = 3) in the same column followed by different superscript letters (a, b) indicate significant differences (*p* < 0.05) between fresh and thermally processed samples of the same extract (Student’s *t*-test-GraphPad Prism Version 8.0.1, Graph Pad Software, Inc., San Diego, CA, United States). WBF: fresh wheat bran; WBTP: thermally processed wheat bran.

**Table 3:** Phenolic compound analysis of the fresh and thermally processed oat bran samples.

Sample	di-OH Benzoic Acid	Caffeic Acid	Vanilic Acid	*p*-Coumaric Acid	Sinapic Acid	Ferulic Acid	Avenantr. 2c	Avenantr. 2p	Avenantr. 2f
**OBF**	55.00 ± 1.02 ^b^	8.61 ± 0.03 ^b^	10.18 ± 0.06 ^b^	2.58 ± 0.03 ^b^	4.67 ± 0.0 ^b^	4.59 ± 0.03 ^b^	4.14 ±0.05 ^b^	7.04 ± 0.02 ^b^	7.85 ±0.09 ^b^
**OBTP**	76.2 ± 1.14 ^a^	22.8 ± 0.19 ^a^	12.48 ± 0.05 ^a^	3 ± 0.03 ^a^	5.28 ± 0.04 ^a^	4.86 ± 0.02 ^a^	6.3 ±0.07^a^	8.69 ± 0.09 ^a^	9.16 ± 0.07 ^a^

Values (mean ± SD, µg/g DW, *n* = 3) in the same column followed by different superscript letters (a, b) indicate significant differences (*p* < 0.05) between fresh and thermally processed samples of the same extract (Student’s *t*-test-GraphPad Prism Version 8.0.1, Graph Pad Software, Inc., San Diego, CA, United States). OBF: fresh oat bran; OBTP: thermally processed oat bran.

**Table 4:** Minimum inhibitory concentration (mg wheat/oat bran/mL) of the WB and OB extracts before and after thermal processing.

Test Items		*Gram (+)*		*Gram (−)*		
		*S. aureus*	*E. faecalis*	*P. aeruginosa*	*E. coli*	*S. thyphimurium*
mg/ML						
WBF	MIC	15	3.75	7.5	15	15
WBTP	MIC	3.75	1.875	3.75	3.75	7.5
OBF	MIC	3.75	7.5	15	30	15
OBTP	MIC	0.9375	3.75	7.5	15	7.5
Streptomycin µg/mL	MIC	0.015	0.12	0.06	0.06	0.015

**Table 5:** Antimutagenic activity toward *S. typhimurium* TA98 and TA100.

Sample	Number of Revertants			
	TA 98		TA100	
	Mean ± SD	Inhibition %	Mean ± SD	Inhibition %
Negative Control	9.45 ± 3.5 ^a^		9.45 ± 1.4 ^a^	
WBF	134 ± 4.7	31.28	174 ± 5.9	50.56
WBTP	121 ± 3.1	37.95	145 ± 5.2	58.84
OBF	87 ± 3.23	55.38	136 ± 3.9	61.36
OBTP	83 ± 2.12	57.43	115 ± 3.1	67.32
4-NPD ^b^	195 ± 10.2	-	-	-
NaN_3_^b^	-	-	352 ± 13.36	-

^a^ Values expressed are means ± S.D. of three replicates. ^b^ 4-nitro-o-phenylenediamine (4-NPD) and NaN_3_ represent the positive controls for *S. thyphimurium* TA98 and TA100 strains, respectively.

## References

[1] Deroover L., Tie Y., Verspreet J., Courtin C.M., Verbeke K. (2019). Modifying wheat bran to improve its health benefits. Crit. Rev. Food Sci. Nutr..

[2] Călinoiu L.F., Vodnar D.C. (2018). Whole Grains and Phenolic Acids: A Review on Bioactivity, Functionality, Health Benefits and Bioavailability. Nutrients.

[3] European Flour Millers. http://www.flourmillers.eu/.

[4] Nutraceuticals and Natural Product Pharmaceuticals-Google Cărți. https://books.google.ro/books?hl=ro&lr=&id=_jOnDwAAQBAJ&oi=fnd&pg=PP1&dq=calinoiu+nutraceuticals&ots=twfYzrgtrC&sig=jhOtSShF8QkKfqNdA38ecLZRJ0&redir_esc=y#v=onepage&q=calinoiu%20nutraceuticals&f=false.

[5] Björck I., Östman E., Kristensen M., Mateo Anson N., Price R.K., Haenen G.R.M.M., Havenaar R., Bach Knudsen K.E., Frid A., Mykkänen H. (2012). Cereal grains for nutrition and health benefits: Overview of results from in vitro, animal and human studies in the HEALTHGRAIN project. Trends Food Sci. Technol..

[6] Călinoiu L.F., Cătoi A.-F., Vodnar D.C. (2019). Solid-State Yeast Fermented Wheat and Oat Bran as A Route for Delivery of Antioxidants. Antioxidants.

[7] Gangopadhyay N., Hossain M.B., Rai D.K., Brunton N.P. (2015). A Review of Extraction and Analysis of Bioactives in Oat and Barley and Scope for Use of Novel Food Processing Technologies. Molecules.

[8] Andersson A.A.M., Dimberg L., Åman P., Landberg R. (2014). Recent findings on certain bioactive components in whole grain wheat and rye. J. Cereal Sci..

[9] Coman V., Teleky B.-E., Mitrea L., Martău G.A., Szabo K., Călinoiu L.-F., Vodnar D.C., Toldrá F. (2019). Bioactive potential of fruit and vegetable wastes. Advances in Food and Nutrition Research.

[10] Călinoiu L.F., Mitrea L., Precup G., Bindea M., Rusu B., Szabo K., Dulf F.V., Ştefănescu B.E., Vodnar D.C., Springer S., Grimm H. (2018). Sustainable use of agro-industrial wastes for feeding 10 billion people by 2050. Professionals in Food Chains.

[11] Szabo K., Dulf F.V., Diaconeasa Z., Vodnar D.C. (2019). Antimicrobial and antioxidant properties of tomato processing byproducts and their correlation with the biochemical composition. LWT.

[12] Trif M., Vodnar D.C., Mitrea L., Rusu A.V., Socol C.T. (2019). Design and Development of Oleoresins Rich in Carotenoids Coated Microbeads. Coatings.

[13] Mitrea L., Calinoiu L.-F., Precup G., Bindea M., Rusu B., Trif M., Stefanescu B.-E., Pop I.-D., Vodnar D.-C. (2017). Isolated Microorganisms for Bioconversion of Biodiesel-Derived Glycerol Into 1,3-Propanediol. Bull. Univ. Agric. Sci. Vet. Med. Cluj-Napoca-Food Sci. Technol..

[14] Vodnar D.C., Venus J., Schneider R., Socaciu C. (2010). Lactic Acid Production by Lactobacillus paracasei 168 in Discontinuous Fermentation Using Lucerne Green juice as Nutrient Substitute. Chem. Eng. Technol..

[15] Szabo K., Cătoi A.-F., Vodnar D.C. (2018). Bioactive Compounds Extracted from Tomato Processing by-Products as a Source of Valuable Nutrients. Plant Foods Hum. Nutr..

[16] Kim K., Tsao R., Yang R., Cui S. (2006). Phenolic acid profiles and antioxidant activities of wheat bran extracts and the effect of hydrolysis conditions. Food Chem..

[17] Coman V., Vodnar D.C. (2020). Hydroxycinnamic acids and human health: Recent advances. J. Sci. Food Agric..

[18] Chen C.-Y.O., Milbury P.E., Collins F.W., Blumberg J.B. (2007). Avenanthramides Are Bioavailable and Have Antioxidant Activity in Humans after Acute Consumption of an Enriched Mixture from Oats. J. Nutr..

[19] Laddomada B., Caretto S., Mita G. (2015). Wheat Bran Phenolic Acids: Bioavailability and Stability in Whole Wheat-Based Foods. Molecules.

[20] Vitaglione P., Napolitano A., Fogliano V. (2008). Cereal dietary fibre: A natural functional ingredient to deliver phenolic compounds into the gut. Trends Food Sci. Technol..

[21] Zhang J., Ding Y., Dong H., Hou H., Zhang X. (2018). Distribution of Phenolic Acids and Antioxidant Activities of Different Bran Fractions from Three Pigmented Wheat Varieties. J. Chem..

[22] Verma B., Hucl P., Chibbar R.N. (2008). Phenolic Content and Antioxidant Properties of Bran in 51 Wheat Cultivars. Cereal Chem..

[23] Chen Y., Zhang R., Liu C., Zheng X., Liu B. (2016). Enhancing antioxidant activity and antiproliferation of wheat bran through steam flash explosion. J. Food Sci. Technol..

[24] Zhang M., Chen H., Li J., Pei Y., Liang Y. (2010). Antioxidant properties of tartary buckwheat extracts as affected by different thermal processing methods. LWT-Food Sci. Technol..

[25] Li W., Pickard M.D., Beta T. (2007). Effect of thermal processing on antioxidant properties of purple wheat bran. Food Chem..

[26] Ragaee S., Seethraman K., Abdel-Aal E.-S.M., de Pinho Ferreira Guiné R., dos Reis Correia P.M. (2016). Effects of Processing on Nutritional and Functional Properties of Cereal Products. Engineering Aspects of Cereal and Cereal-Based Products.

[27] Vodnar D.C., Călinoiu L.F., Dulf F.V., Ştefănescu B.E., Crişan G., Socaciu C. (2017). Identification of the bioactive compounds and antioxidant, antimutagenic and antimicrobial activities of thermally processed agro-industrial waste. Food Chem..

[28] Calinoiu L.-F., Mitrea L., Precup G., Bindea M., Rusu B., Dulf F.-V., Stefanescu B.-E., Vodnar D.-C. (2017). Characterization of Grape and Apple Peel Wastes’ Bioactive Compounds and Their Increased Bioavailability After Exposure to Thermal Process. Bull. Univ. Agric. Sci. Vet. Med. Cluj-Napoca-Food Sci. Technol..

[29] Dewanto V., Wu X., Liu R.H. (2002). Processed Sweet Corn Has Higher Antioxidant Activity. J. Agric. Food Chem..

[30] Ștefănescu B.E., Szabo K., Mocan A., Crişan G. (2019). Phenolic Compounds from Five Ericaceae Species Leaves and Their Related Bioavailability and Health Benefits. Molecules.

[31] Pasqualone A., Delvecchio L.N., Gambacorta G., Laddomada B., Urso V., Mazzaglia A., Ruisi P., Di G.M. (2015). Effect of Supplementation with Wheat Bran Aqueous Extracts Obtained by Ultrasound-Assisted Technologies on the Sensory Properties and the Antioxidant Activity of Dry Pasta. Nat. Prod. Commun..

[32] Pasqualone A., Gambacorta G., Summo C., Caponio F., Di Miceli G., Flagella Z., Marrese P.P., Piro G., Perrotta C., De Bellis L. (2016). Functional, textural and sensory properties of dry pasta supplemented with lyophilized tomato matrix or with durum wheat bran extracts produced by supercritical carbon dioxide or ultrasound. Food Chem..

[33] Calinoiu L.-F., Vodnar D.-C., Precup G. (2016). The Probiotic Bacteria Viability under Different Conditions. Bull. Univ. Agric. Sci. Vet. Med. Cluj-Napoca-Food Sci. Technol..

[34] Călinoiu L.-F., Ştefănescu B.E., Pop I.D., Muntean L., Vodnar D.C. (2019). Chitosan Coating Applications in Probiotic Microencapsulation. Coatings.

[35] Martău G.A., Mihai M., Vodnar D.C. (2019). The Use of Chitosan, Alginate, and Pectin in the Biomedical and Food Sector—Biocompatibility, Bioadhesiveness, and Biodegradability. Polymers.

[36] Vasile C., Rapa M., Stefan M., Stan M., Macavei S., Darie-Nita R.N., Barbu-Tudoran L., Vodnar D.C., Popa E.E., Stefan R. (2017). New PLA/ZnO:Cu/Ag bionanocomposites for food packaging. Express Polym. Lett..

[37] Vodnar D.C., Socaciu C. (2012). Green tea increases the survival yield of Bifidobacteria in simulated gastrointestinal environment and during refrigerated conditions. Chem. Cent. J..

[38] Bryngelsson S., Dimberg L.H., Kamal-Eldin A. (2002). Effects of Commercial Processing on Levels of Antioxidants in Oats. J. Agric. Food Chem..

[39] Wang T., He F., Chen G. (2014). Improving bioaccessibility and bioavailability of phenolic compounds in cereal grains through processing technologies: A concise review. J. Funct. Foods.

[40] Ma Y., Kosińska-Cagnazzo A., Kerr W.L., Amarowicz R., Swanson R.B., Pegg R.B. (2014). Separation and characterization of phenolic compounds from dry-blanched peanut skins by liquid chromatography–electrospray ionization mass spectrometry. J. Chromatogr. A.

[41] Dulf F.V., Vodnar D.C., Dulf E.-H., Toşa M.I. (2015). Total Phenolic Contents, Antioxidant Activities, and Lipid Fractions from Berry Pomaces Obtained by Solid-State Fermentation of Two Sambucus Species with Aspergillus niger. J. Agric. Food Chem..

[42] Dulf F.V., Vodnar D.C., Socaciu C. (2016). Effects of solid-state fermentation with two filamentous fungi on the total phenolic contents, flavonoids, antioxidant activities and lipid fractions of plum fruit (*Prunus domestica L*.) byproducts. Food Chem..

[43] Dezsi Ș., Bădărău A.S., Bischin C., Vodnar D.C., Silaghi-Dumitrescu R., Gheldiu A.-M., Mocan A., Vlase L. (2015). Antimicrobial and antioxidant activities and phenolic profile of *Eucalyptus globulus Labill. and Corymbia ficifolia* (F. Muell.) K.D. Hill & L.A.S. Johnson leaves. Mol. Basel Switz..

[44] Toma C.-C., Olah N.-K., Vlase L., Mogoșan C., Mocan A. (2015). Comparative Studies on Polyphenolic Composition, Antioxidant and Diuretic Effects of *Nigella sativa L.* (Black Cumin) and *Nigella damascena L.* (Lady-in-a-Mist) Seeds. Mol. Basel Switz..

[45] Maron D.M., Ames B.N. (1983). Revised methods for the Salmonella mutagenicity test. Mutat. Res..

[46] Saraç N., Şen B. (2014). Antioxidant, mutagenic, antimutagenic activities, and phenolic compounds of *Liquidambar orientalis Mill. var. orientalis*. Ind. Crop. Prod..

[47] Ong T.M., Whong W.Z., Stewart J., Brockman H.E. (1986). Chlorophyllin: A potent antimutagen against environmental and dietary complex mixtures. Mutat. Res..

[48] Evandri M.G., Battinelli L., Daniele C., Mastrangelo S., Bolle P., Mazzanti G. (2005). The antimutagenic activity of *Lavandula angustifolia* (lavender) essential oil in the bacterial reverse mutation assay. Food Chem. Toxicol..

[49] Wang J., Sun B., Cao Y., Tian Y., Li X. (2008). Optimisation of ultrasound-assisted extraction of phenolic compounds from wheat bran. Food Chem..

[50] Sharanappa T., Chetana R., Suresh Kumar G. (2016). Evaluation of genotypic wheat bran varieties for nutraceutical compounds. J. Food Sci. Technol..

[51] Brindzová L., Zalibera M., Jakubík T., Mikulášová M., Takácsová M., Mošovská S., Rapta P. (2009). Antimutagenic and Radical Scavenging Activity of Wheat Bran. Cereal Res. Commun..

[52] Chandrasekara A., Naczk M., Shahidi F. (2012). Effect of processing on the antioxidant activity of millet grains. Food Chem..

[53] Dai J., Mumper R.J. (2010). Plant phenolics: Extraction, analysis and their antioxidant and anticancer properties. Mol. Basel Switz..

[54] Norton T., Sun D.-W. (2008). Recent Advances in the Use of High Pressure as an Effective Processing Technique in the Food Industry. Food Bioprocess. Technol..

[55] Hostetler G.L., Ralston R.A., Schwartz S.J. (2017). Flavones: Food Sources, Bioavailability, Metabolism, and Bioactivity12. Adv. Nutr..

[56] Adom K.K., Sorrells M.E., Liu R.H. (2005). Phytochemicals and Antioxidant Activity of Milled Fractions of Different Wheat Varieties. J. Agric. Food Chem..

[57] Yu L.L. (2008). Wheat Antioxidants.

[58] Cheng Z., Su L., Moore J., Zhou K., Luther M., Yin J.-J., Yu L. (2006). (Lucy) Effects of Postharvest Treatment and Heat Stress on Availability of Wheat Antioxidants. J. Agric. Food Chem..

[59] Ragaee S., Seetharaman K., Abdel-Aal E.-S.M. (2014). The impact of milling and thermal processing on phenolic compounds in cereal grains. Crit. Rev. Food Sci. Nutr..

[60] Daniels D.G.H., Martin H.F. (1968). Antioxidants in oats: Glyceryl esters of caffeic and ferulic acids. J. Sci. Food Agric..

[61] Cai S., Wang O., Wu W., Zhu S., Zhou F., Ji B., Gao F., Zhang D., Liu J., Cheng Q. (2012). Comparative Study of the Effects of Solid-State Fermentation with Three Filamentous Fungi on the Total Phenolics Content (TPC), Flavonoids, and Antioxidant Activities of Subfractions from Oats (*Avena sativa L*.). J. Agric. Food Chem..

[62] Durkee A.B., Thivierge P.A. (1977). Ferulic Acid And Other Phenolics In Oat Seeds (*Avena sativa L. Var Hinoat*). J. Food Sci..

[63] Shewry P.R., Piironen V., Lampi A.-M., Nyström L., Li L., Rakszegi M., Fraś A., Boros D., Gebruers K., Courtin C.M. (2008). Phytochemical and Fiber Components in Oat Varieties in the HEALTHGRAIN Diversity Screen. J. Agric. Food Chem..

[64] Dimberg L.H., Sunnerheim K., Sundberg B., Walsh K. (2001). Stability of Oat Avenanthramides. Cereal Chem..

[65] Thermal Decomposition of Ferulic Acid. https://pubs.acs.org/doi/pdf/10.1021/jf60153a003.

[66] Brindzová L., Mikulášová M., Takácsová M., Mošovská S., Opattová A. (2009). Evaluation of the mutagenicity and antimutagenicity of extracts from oat, buckwheat and wheat bran in the Salmonella/microsome assay. J. Food Compos. Anal..

[67] Birošová L., Mikulášová M., Vaverková S. (2005). Antimutagenic effect of phenolic acids-Google Academic Biomed. Pap Med Fac Univ Palacky Olomouc Czech Repub..

[68] Wu S.-C., Yen G.-C., Wang B.-S., Chiu C.-K., Yen W.-J., Chang L.-W., Duh P.-D. (2007). Antimutagenic and antimicrobial activities of pu-erh tea. LWT-Food Sci. Technol..

